# Supplementary material for: Use of Antivibration Technology to Reduce Demands for In-Home Nursing Care and Support in Rural Settings for Persons with Essential Tremors: A Qualitative Study
Source: Int J Environ Res Public Health. 2024 May 31;21(6):714. doi: 10.3390/ijerph21060714 (PMC11203956; doi:10.3390/ijerph21060714)
Supplement: Supplementary file 1 [file ijerph-21-00714-s001.zip › ijerph-2912934-supplementary.pdf]

Supplemental File S1. Codes and Main Themes that Emerged from Data Analysis

Codes	Themes and subthemes
**1.** Nursing shortage	
**2.** Living in rural areas	
**3.** Lack of instructions and trainings	
**4.** Long hour shifts	
**5.** The structure of intra-organizational technologies	
**6.** Registered Nurses playing their roles based on the various tasks defined in the organization	**1.** Registered Nurses' views on using technology in their nursing practice
**7.** Extra workload	
**8.** Various organizations; various structures; different technologies	
**9.** Assimilating the RNs, especially the newcomers	1a. The integration between technology and nursing
**10.** The effects of Information and Communication Technologies on RNs' workload	1b. Registered Nurses' expectation of future technologies for reducing their workload
**11.** Registered Nurses' experiences working with Information and Communication Technologies	1c. Registered Nurses' attitude towards learning a new technology
**12.** The effects of E-learning on RNs' workload	
**13.** Registered Nurses' experiences working with Information and Communication Technologies	
**14.** Registered Nurses' attitudes towards Steadiwear gloves	
**15.** Registered Nurses' experiences working with E-learning	
**16.** The effects of mHealth on RNs' workload	
**17.** The effect of Steadi-One gloves on the independence of essential tremors patients	**2.** Factors affecting technology use by RNs
**18.** The effects of E-communications on RNs' workload	
**19.** Registered Nurses' experiences	2a. Decision making based

working with E-communications	→ on urban setting
**20.** The effects of Assistive Devices on RNs' workload	→ 2b. Technology resources
**21.** Registered Nurses' experiences working with Assistive Devices	→ 2c. Suitable training and support for using technology
**22.** Registered Nurses' experiences working with mHealth	
